# Remembering to Care for Each Other

**DOI:** 10.1016/j.jscai.2025.102578

**Published:** 2025-03-04

**Authors:** Robert F. Riley, Nishtha Sareen

**Affiliations:** a Overlake Medical Center and Clinics, Bellevue, Washington; b Ascension Saint Thomas Heart, Nashville, Tennessee

**Keywords:** cath laboratory, occupational health, radiation

There is an old adage that is routinely taught at the beginning of internship—“You can’t take care of others unless you take care of yourself.” This has been passed down by mentors to trainees in order to promote a healthy balance during the rigors of medical training. We pass on similar recommendations to new mothers and significant others of the acutely ill; however, somewhere along the way, this advice can get lost in the business of balancing medical practice with the demands of life outside the hospital, leaving many of us less healthy than when we started.

In this issue of *JSCAI*, Abudayyeh et al^1^ present the results of a survey developed by the SCAI Professional Well-Being Committee regarding the occupational health hazards of working in the cardiac catheterization laboratory. The survey specifically keyed in on orthopedic, cancer, and pregnancy-related risks of routine exposure to continuous fluoroscopy and the wearable lead used to mitigate this risk. These data were collected in 2023 among 296 respondents and were compared with similar data collected in 2014.

Unsurprisingly, the survey found that the vast majority of respondents (82%) experienced musculoskeletal pain and/or orthopedic injuries from working in the catheterization laboratory, with roughly 20% having to limit their time in the catheterization laboratory due to injury and/or need to reduce radiation exposure. This risk increased with age and showed a higher burden in older and more experienced respondents. This prevalence was substantially higher than in the general population (∼47% prevalence).^2^ Additionally, 6% of respondents reported a diagnosis of cancer, 5% reported cataracts, and 5% had various forms of skin disease, although higher numbers reported knowing someone else that works in a catheterization laboratory with these diseases.

While attributable risk from continuous radiation exposure for these diseases is difficult to ascertain, the elevated prevalence of these diseases compared with the general public certainly highlights the substantial risk that ionizing radiation can have on health care workers.^3^, ^4^, ^5^, ^6^ Finally, a majority of female respondents reported that they either had significant concerns about working in the catheterization laboratory while pregnant or were discouraged by their institution from working in the catheterization laboratory while pregnant.

While today’s modern catheterization laboratory facilitates a wide range of diagnostic and therapeutic procedures, the prolonged and repeated use of continuous ionizing radiation during these procedures raises significant safety concerns for both patients and health care personnel despite the use of standard lead shielding. With the increasing complexity of catheter-based interventions and expansion into the structural space, the importance of radiation safety for health care workers has become even more paramount. Although lead shields and aprons are often thought of as radiation barriers, they act more as filters, depending on the “lead equivalency” (or strength) of the shield.^7^ In addition to their imperfect radiation protection, wearable lead aprons result in significant orthopedic injuries with longitudinal use due to their heavy weight and imperfect ergonomics.^8^ While the orthopedic burden of working in the catheterization laboratory does not appear to have changed since the last survey in 2014, it remains significant, affecting most interventional cardiologists at 1 or more points in their career. This limits their ability to provide care during absences due to ongoing pain or disability, associated illnesses, and the ramifications of dealing with chronic pain, not to mention the long-term consequence of these injuries for physicians and catheterization laboratory staff. While many respondents reported various types of physical exercise and/or therapy to treat chronic musculoskeletal pain/injuries once present, utilization of novel radiation protection systems to prevent these types of injuries was low. Cost/lack of administrative buy-in was cited as a major source of low utilization rates for these devices.

We congratulate the authors on highlighting the critical issue of needing to improve radiation safety in the catheterization laboratory. The acknowledged risks of working in the laboratory have been minimized for far too long, and we have seen too many of our colleagues experience the ramifications of exposure to x-ray scatter and/or long-term use of lead aprons. Additionally, concerns cited by female respondents about radiation exposure is consistent with a recent survey of fellows-in-training citing this as a major concern when considering pursuing a career in the catheterization/electrophysiology laboratory.^9^ While enhanced radiation protection in the catheterization laboratory is only a part of efforts to address the gender divide in interventional cardiology, we must continue to find ways to mitigate these real concerns.

**Figure 1:**
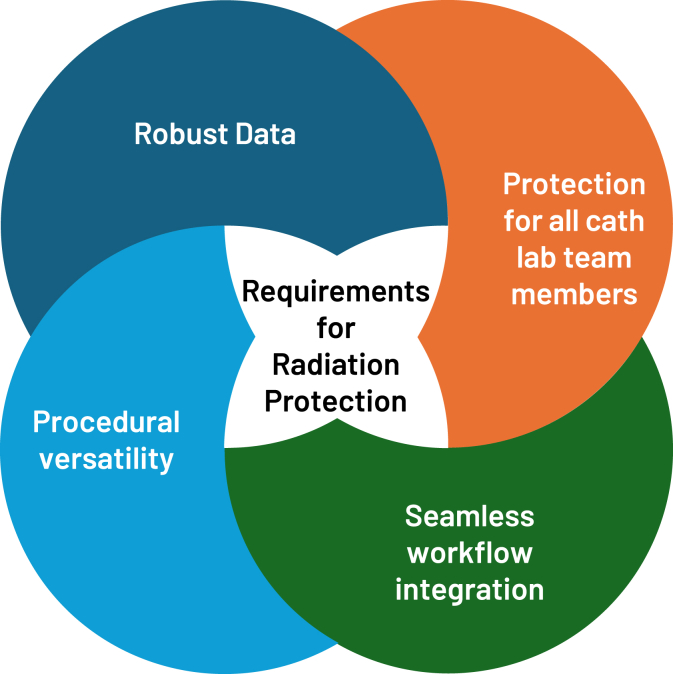
**Attributes of ideal radiation protection systems**.

There are several novel radiation protection devices (eg, EggNest [Egg Medical], Rampart [RAMPART ic], and Protego [Image Diagnostics])^10^^,^^11^ that can help significantly reduce exposure to ionizing radiation scatter and the need for heavy lead aprons in the catheterization laboratory. While more of these devices may come to market in the coming years, several key components are necessary to ensure they provide adequate protection, including: robust data regarding efficacy, ability to shield everyone in the room (not just the primary operator[s]), versatility in utilization (eg, interventional radiology, catheterization laboratory, structural, electrophysiology, vascular surgery, and emergency procedures), and seamless integration into work flow (Figure 1).

Unfortunately, lack of administrative buy-in was cited as a major source of low utilization rates for these devices. It is time for our community to demand further radiation safety under the principle of ALARA (as low as reasonably achievable), with the goal of eliminating a barrier for some of our colleagues from practicing in the laboratory and reducing the significant longitudinal health impacts on catheterization laboratory personnel. We are excited about SCAI’s upcoming efforts to promote work-related safety for its members and look forward to both innovation in this field and further uptake of novel radiation protection technology.
